# Labral Reconstruction: When to Perform and How

**DOI:** 10.3389/fsurg.2015.00027

**Published:** 2015-07-02

**Authors:** Brian J. White, Mackenzie M. Herzog

**Affiliations:** ^1^Western Orthopaedics, Denver, CO, USA; ^2^Professional Research Institute for Sports Medicine, LLC, Chapel Hill, NC, USA

**Keywords:** surgical techniques, hip arthroscopy, labral tear, labral reconstruction, labral pathology

## Abstract

Over the past decade, the understanding of the anatomy and function of the hip joint has continuously evolved, and surgical treatment options for the hip have significantly progressed. Originally, surgical treatment of the hip primarily involved resection of damaged tissue. Procedures that maintain and preserve proper hip anatomy, such as labral repair and femoroacetabular impingement correction, have shown superior results, in terms of pain reduction, increased function, and ability to return to activities. Labral reconstruction is a treatment option that uses a graft to reconstruct the native labrum. The technique and outcomes of labral reconstruction have been described relatively recently, and labral reconstruction is a cutting edge procedure that has shown promising early outcomes. The aim of this article is to review the current literature on hip labral reconstruction. We will review the indications for labral reconstruction, surgical technique and graft options, and surgical outcomes that have been described to date. Labral reconstruction provides an alternative treatment option for challenging intra-articular hip problems. Labral reconstruction restores the original anatomy of the hip and has the potential to preserve the longevity of the hip joint. This technique is an important tool in the orthopedic surgeon’s arsenal for hip joint treatment and preservation.

## Background

Hip arthroscopy is a relatively new frontier in orthopedic surgery, with the first documented arthroscopy of the hip performed less than a century ago in 1931 (1, 2). Originally, arthroscopic treatment of the hip primarily involved diagnosis or, at most, irrigation, or simple resection of damaged tissue (1). Since that time, the understanding of the anatomy and function of the hip has continuously evolved, and surgical treatment options have significantly progressed to include a multitude of different procedures (1, 3).

Labral pathology is one of the most common diagnoses among adolescent and adult patients who present for treatment of hip pain (4, 5). The estimated prevalence of labral pathology is not well understood, but previous reports range from 22 to 55% in clinical population (4, 6, 7). Although the prevalence is not well understood, the understanding of the role of the acetabular labrum to biomechanical functioning of the hip has improved significantly in recent years. The labrum plays a crucial role in the stability, lubrication, and kinematics of the hip (8–15). Consequently, surgical procedures that maintain and preserve proper hip anatomy, such as labral repair (versus labral debridement) and FAI correction, have shown superior results in comparison, in terms of pain reduction, increased function, and ability to return to activities (5, 8–10, 16–19).

Labral reconstruction was first described in 2009 and is a treatment option that uses a graft to reconstruct the native labrum (20). The technique and outcomes of labral reconstruction have been described relatively recently, and labral reconstruction is a cutting edge procedure that has shown promising early outcomes. The aim of this article is to review the current literature on hip labral reconstruction. We will review the indications for labral reconstruction, surgical technique and graft options, and outcomes that have been described to date.

## Indications for Surgery

### Biomechanical advantages

The labrum plays an important role in maintaining normal hip function. A previous cadaveric study indicated that partial labral resection resulted in loss of fluid pressurization and change of the hip seal (10). Labral reconstruction not only improved fluid pressurization, but maintained it over time, even better than labral repair in that study (10). While early biomechanical research supports labral reconstruction overall, one study does suggest that labral reconstruction may not prevent fluid efflux compared to labral repair or intact labral state (21).

The acetabular labrum also plays an important role in stabilization of the joint to distraction forces (9). Similar to the study of hip fluid pressurization, labral reconstruction was found to significantly improve stability to distractive forces compared to partial labral resection (9).

More recently, a cadaveric study assessed the contact area, contact pressure, and peak force in hips with labral pathology compared to hips with a reconstructed acetabular labrum (15). Hip contact pressure increased in the presence of labral resection but was reduced with labral reconstruction (15). In addition, labral reconstruction reduced peak forces in the hip compared to labral resection. These studies suggest that certain types of labral pathology may be indicated for labral reconstruction.

### Patient characteristics

Arthroscopic labral repair has shown promising patient outcomes (22); however, there exists a population of patients in which labral repair is less optimal. The primary indications for labral reconstruction include irreparable labral tears or insufficient labral tissue (Table S1 in Supplementary Material) (8, 18, 20, 23–27). In these cases, a labral repair may not be feasible or adequate to restore the fluid seal of the hip joint (25, 26). When the tissue is too small, it lacks surface area to heal or the repair may not provide an adequate seal with the femoral head (9, 10). For this reason, a labrum <2–3 mm is considered an indication for labral reconstruction (28). On the contrary, when the tissue is too large, compression often cannot be achieved to allow the labrum to heal. Therefore, a labrum >8 mm is considered an indication for labral reconstruction, although this threshold has not been formally established in the literature. Labral tissue that is degenerative with intrasubstance cystic degeneration is also an indication for reconstruction. Revision procedures following previous labral debridement or resection often provide a challenging situation in which adequate labral tissue may not be available (18, 27). Labral reconstruction provides a viable alternative for maintaining and preserving labral function in patients with irreparable labral tears or insufficient labral tissue for repair.

Labral reconstruction may also be indicated for a variety of other reasons. In the presence of capsulolabral adhesions from previous surgery, it may not be possible to excise the scar tissue while preserving enough labral tissue to repair (25). Patients with rim ossification or global over coverage of the acetabulum may also benefit from labral reconstruction (8, 20, 23). Although contraindications for labral reconstruction have not been well-described, older patient age and preoperative joint space ≤2 mm have been proposed (Table S1 in Supplementary Material) (25). Overall, labral reconstruction should be considered in cases where the ability to maintain and preserve the native hip anatomy is compromised.

## Surgical Technique

### Open technique

Sierra and Trousdale first reported hip labral reconstruction in 2009 (20). The original technique was described in association with an open surgical hip dislocation (20). Briefly, the technique described use of a ligamentum teres capitis autograft. The ligamentum teres was detached from the fovea and fixed to the acetabular rim in the same manner as labral refixation. In cases where the size of the ligamentum teres was not sufficient to adequately reconstruct the labrum, the ligament was opened longitudinally in order to lengthen the graft.

Open surgical dislocation remains an option for patients who meet indications for reconstruction. However, in recent years, hip arthroscopy has emerged as a new, less invasive treatment. While once considered the gold standard for surgical treatment of the hip, several recent studies have questioned the superiority of open surgical dislocation to arthroscopy (29–32). Although randomized comparative studies of open and arthroscopic techniques are lacking, hip arthroscopy has significantly fewer complications and re-operations versus open surgical dislocation (29–32).

### Arthroscopic technique

Several arthroscopic techniques for labral reconstruction have been previously described (18, 27, 33–35). A modification of the original arthroscopic technique is presented here, including a front-to-back fixation technique (18, 28), and publication of the technique is currently in press (36). Briefly, the procedure is performed with the patient in a supine position on a fracture table. Combined general and spinal anesthesia is used, with an epidural anesthetic utilized in younger patients (<20 years). Rocuronium, a heavy paralytic agent, is employed at a loading dose of 1.5 mg per 1 kg. Total traction time does not exceed 90 min (in ≤45 min intervals).

Three arthroscopic portals are created for this procedure, including an anterolateral, mid anterior, and accessory portal. Three portals are necessary to maintain appropriate graft tension throughout the procedure. The anterolateral portal is located slightly anterior to the superior tip of the greater trochanter. The mid anterior portal is located 6 cm medial to the anterolateral portal and roughly 1 cm distal. The third, accessory portal is placed roughly 2–3 cm distal and 1–2 cm posterior to the mid anterior portal.

A femoral osteoplasty is performed to correct head-neck offset to eliminate any cam impingement and provide an excellent bleeding environment for graft incorporation. The acetabular rim is also reshaped in order to establish an improved anatomic shape, remove the pincer lesion, and expose a flat, congruent bleeding surface on the acetabular rim for graft incorporation. Torn and damaged labral tissue are fully excised from the low anterior portion of the acetabulum at the origin of the transverse acetabular ligament (7:30 on the left hip and 4:30 on right) to the posterior aspect of the acetabulum (typically 3:00 on the left hip and 9:00 on the right hip). No native labral tissue is retained in the anterior quadrant of the acetabulum because it is felt that loss of connection to the circumferential labrum leads to loss of hoop strength.

In preparation for graft placement, the labral defect is measured, and the graft length is overestimated to avoid the graft being too short. Anchors are placed close to the acetabular edge, without breeching the joint, roughly 11–14 mm apart. They are inserted from the distal accessory portal into the acetabular rim. The most antero-inferior anchor is placed as close to the origin of the anterior transverse acetabular ligament as possible.

**Figure 1:**
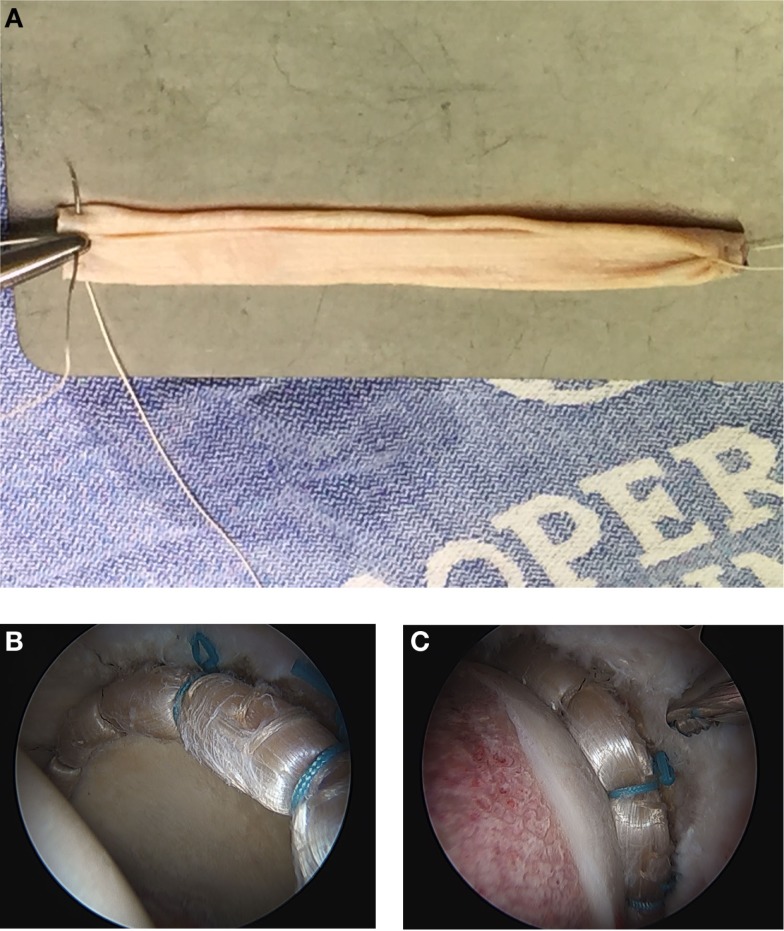
**(A)** The iliotibial band allograft is measured and rolled to create a final tubularized graft, measuring roughly 5–6 mm in diameter. The graft is folded into thirds and a 2-0 Vicryl suture is placed at each end of the graft using an accordion-type suture technique, where several small bites are taken across the end of the graft. (**B**) After the labral reconstruction, this is a view of a left hip from the anterolateral portal, showing an 11 cm labral reconstruction using iliotibial band allograft and nine anchors in traction. (**C**) After the labral reconstruction, this is a view of a left hip from the anteromedial portal showing an 11 cm labral reconstruction using iliotibial band allograft and nine anchors. The joint is reduced with a view of the re-established seal between the reconstructed labrum and the femoral head.

For this technique, an iliotibial band allograft (AlloSource, Centennial, CO, USA and MTF Sports Medicine, Edison, NJ, USA), freeze dried or frozen, is preferred. The graft is prepared on the back table by soaking it in a 250 cc saline and 80 mg Gentamycin solution. Once thawed, the graft is measured and rolled to create a final tubularized graft measuring roughly 5–6 mm in diameter. The graft is folded into thirds and a 2-0 Vicryl suture is placed at each end of the graft using an accordion-type suture technique, where several small bites are taken across the end of the graft (Figure 1A). When tied, the tension bunches the graft and begins the tubularization process. Each suture is secured in the Graftmaster and another 2-0 absorbable Vicryl suture is run up and down the length of the graft to tubularize the graft (18).

The cannula is placed from the distal accessory portal through the intact antero-inferior capsule. The graft is then introduced into the joint, by fixing it to the non-post end of the first suture and pulling it into the joint with the post end. The suture is then tied with alternating half hitch sutures once the graft is provisionally placed in an appropriate position along the rim of the acetabulum. Circumferential sutures are tied from the distal accessory portal working from anterior to posterior. The second to last suture is passed but not tied to allow for mobility at the end of the graft. Excess graft is removed by cutting the graft with a beaver blade from the anterolateral portal, while maintaining tension with a grasper from the distal accessory portal. The most posterior suture is passed through the end of the graft with an ElitePass (Smith & Nephew, London, England) and then passed under and around the graft, creating a Mason-Allen type of suture construct. The graft is inspected after traction is taken down to ensure there is a complete, continuous seal between the graft and the femoral head (Figures 1B,C). Dynamic testing of the joint is done to assess the shape of the joint and to ensure there is no impingement. To complete the procedure, the anterior portion of the capsule is closed.

### Graft options

Several graft options for labral reconstruction have been previously proposed. Allograft tissue provides several benefits over autograft tissue, including the ability to control graft thickness, length, and consistency and the ability to eliminate donor site morbidity. In addition, allograft tissue is aneural, providing a potential benefit in pain reduction. In contrast, native labral tissue likely remains innervated during and following the repair process, which could lead to future pain. Other proposed graft options include iliotibal band autograft (18, 27), local capsular autograft (33), gracilis autograft (34), ligamentum teres capitis autograft (20), fascia lata autograft (23), and quadriceps tendon autograft (35). Advantages and disadvantages of these graft options have not been thoroughly explored in existing literature at this time; however, a recent study of comparing biomechanical properties of the native labrum to iliotibial band, gracilis, semitendinosus, and anterior tibialis grafts found similar biomechanical properties but differing levels of variability in elongation and geometry (37). Another recent study compared iliotibial band autograft to semitendinosus allograft and found no significant difference in contact area, contact pressure, or peak force (15).

### Postoperative management

Rehabilitation for labral reconstruction is similar to that for labral repair; however, patients are cautioned regarding the aneural properties of their graft. Patients begin supervised physical therapy within 1 week of surgery. The focus of rehabilitation is gaining motion, strengthening the gluteus medius, rebalancing the hip musculature, and establishing a normal gait pattern. Weight bearing is typically restricted to 30% body weight for 4 weeks or 20% body weight for 6 weeks if a concomitant microfracture procedure is performed. Advanced rehabilitation focuses on building strength and returning to sport and activity. In our experience, full recovery typically occurs approximately 6 months postoperatively.

## Outcomes

### Open labral reconstruction outcomes

Two published studies have analyzed the results of labral reconstruction performed during open surgical dislocation (Table 1) (20, 23). The first study of outcomes accompanied the original report of the open technique for labral reconstruction (20). The second published study was a case series of 20 hips that underwent labral reconstruction using ligamentum teres capitis or fascia lata autograft in conjunction with FAI treatment (23). The authors reported no complications in either study; however, at a minimum of 1-year follow-up (mean: 26.4 months), 13 hips (65%) underwent 19 subsequent operations, including removal of hardware (*n* = 12), lysis of adhesions (*n* = 2), iliopsoas release (*n* = 1), unspecified arthroscopy (*n* = 1), and THA (*n* = 3) (23).

**Table 1:** **Published open and arthroscopic labral reconstruction outcomes**.

Study	Open vs arthroscopic/graft	*n*	Sex	Age	Follow-up	Convert to THA	Preoperative outcome	Postoperative outcome
Sierra and Trousdale (20)	Open/ligamentum teres capitis autograft	5	3 M, 2 F	33 (19–50) years	10 (5–20) months	1 (20%)	– 3 “severe pain” – 2 “moderately severe pain” – UCLA:5 (2– 6)	– 3 “no pain” – 1 “moderate pain” – 1 “same pain as preoperatively” – UCLA: 8 (6–10)

Walker et al. (23)	Open/ligamentum teres capitis autograft or fascia lata autograft	20	5 M, 14 F	29 (16–50) years	26 (12–56) months	3 (15%)	Not reported	– UCLA: 8.5 (5–10)

White et al. (38)	Arthroscopic/iliotibial band allograft	152	64 M, 78 F	39 (16–58) years	28 (24–39) months	13 (10%)	– MHHS: 54 – LEFS: 41 – VAS rest: 5 – VAS ADLs: 6 – VAS sport: 8	– MHHS: 88 – LEFS: 68 – VAS rest: 2 – VAS ADLs: 2 – VAS sport: 3 – Satisfaction: 9/10

Philippon et al. (18)	Arthroscopic/iliotibial band autograft	47	32 M, 15 F	37 (18–55) years	18 (12–32) months	4 (9%)	– MHHS: 62	– MHHS: 85 – Satisfaction: 8/10

Geyer et al. (25)	Arthroscopic/iliotibial band autograft	76	42 M, 33 F	39 (18–64) years	49 (36–70) months	18 (24%) + 1 (1%) resurface	– MHHS: 59 – HOS-ADL: 69 – HOS-Sport: 41 – SF-12 physical:42 – SF-12 mental:55	– MHHS: 83 – HOS-ADL: 81 – HOS-Sport: 67 – SF-12 physical: 50 – SF-12 mental: 53 – Satisfaction: 8/10

Boykin et al. (24)	Arthroscopic/iliotibial band autograft	21	19 M, 0 F	28 (19–41) years	41 (20–74) months	2 (10%)	– MHHS: 67 – HOS-ADL: 77 – HOS-Sport: 56 – SF-12 physical: 44 – SF-12 mental: 49	– MHHS: 84 – HOS-ADL: 85 – HOS-Sport: 77 – SF-12 physical: 51 – SF-12 mental: 54 – Satisfaction: 8/10 – Returned to play: 18 (86%)

Matsuda and Burchette (26)	Arthroscopic/gracilis autograft	8	7 M, 1 F	35 (18–58) years	30 (24–37) months	0 (0%)	– NAHS: 42	– NAHS: 92 – Satisfaction: 7 “high,”1 “moderate”

Domb et al. (39)	Arthroscopic/gracilis tendon autograft	11	7 M, 4 F	33 (18–45) years	26 (24–32) months	0 (0%)	– NAHS: 53 – HOS-ADL: 59 – HOS-Sport: 39 – MHHS: 55 – VAS: 7	– NAHS: 78 – HOS-ADL: 80 – HOS-Sport: 60 – MHHS: 82 – VAS: 3 – Satisfaction: 8/10

*^a^Data are expressed as count (%) or mean (range)*.

*MHHS, modified harris hip score; HOS-ADL, hip outcome score-activities of daily living; HOS-sport, hip outcome score-sports-specific subscale; SF-12 physical, short form-12 physical component; SF-12 mental, short form-12 mental component; NAHS, non-arthritic hip score; VAS, visual analog scale for pain; ADLs, activities of daily living*.

### Arthroscopic labral reconstruction outcomes

Promising patient-reported outcomes and low revision rate have been achieved with arthroscopic labral reconstruction. The lead author has performed over 1,000 labral reconstructions to date (July 2009–March 2015) and, overall, has found superior results with reconstruction compared to repair or debridement in patients with complex intra-articular pathology. Minimum 2-year outcomes from allograft labral reconstruction by the authors are currently in press (38). Among 152 allograft reconstructions, 118 hips were primary reconstructions and 34 were revision reconstructions. One hundred and thirty-one hips were available for follow-up (86%). Thirteen hips (10%) converted to THA and five hips (4%) underwent revision hip arthroscopy at mean 28 months follow-up. Patients who underwent subsequent surgery were found to have significantly lower preoperative MHHS and LEFS, higher VAS pain scores, and were more likely to have undergone previous open dislocation procedure. Of the remaining hips that did not undergo subsequent procedure (*n* = 113), there was significant improvement in MHHS, LEFS, and VAS for pain at rest, with ADLs, and with athletic activities (Table 1) (*p* < 0.0001). Overall patient-reported satisfaction was 9 on a VAS scale from 1 to 10 (10, extremely satisfied). Future studies will identify outcome in specific patient subsets, compare procedures, and report long-term outcome.

A recent literature review identified five additional published reports of outcomes from arthroscopic labral reconstruction (Table 1) (18, 24–26, 39). The original report of arthroscopic labral reconstruction in 2010 included early outcomes following arthroscopic labral reconstruction with iliotibial band autograft (18, 28). The study reported promising early outcomes (Table 1). No complications were reported, and four hips (9%) progressed to THA at a mean follow-up of 18 months. Continued promising clinical results were reported in this patient population at a mean of 49 months (minimum 3 years) postoperatively (Table 1) (25). The proportion of hips that converted to THA increased to 24% (*n* = 18), with one additional hip converting to resurfacing. Identified patient factors associated with conversion to THA were patient age and preoperative joint space ≤2 mm (18, 25). In an additional report in an elite athlete population, the authors found that 18 of 21 athletes were able to return to the elite playing level following surgery, and 17 of those athletes returned to their previous level of performance or better (24).

Outcomes of arthroscopic labral reconstruction with gracilis autograft have also been reported (26, 39). The first study compared a cohort of eight patients who underwent labral reconstruction to a cohort of 46 patients who underwent labral refixation (26). A second study compared a cohort of 11 patients who underwent labral reconstruction to a cohort of 22 matched patients who underwent arthroscopic segmental labral resection (39). No major complications were reported in either study, but two patients who underwent labral reconstruction had pudendal nerve neuropraxias that resolved within 3 months (26). There were no conversions to THA reported (26, 39). Overall, the labral reconstruction group appeared to have better outcomes than both the labral refixation group and the labral resection group (Table 1) (26, 39).

## Conclusion

Labral reconstruction provides an alternative treatment option for challenging intra-articular hip problems. The primary indications for labral reconstruction are irreparable labral tears or insufficient labral tissue. Labral reconstruction provides several biomechanical advantages as a treatment option for labral pathology, including improved fluid pressurization, stabilization of the hip to distractive forces, and reduced contact pressure in the hip joint (9, 10, 15). Labral reconstruction should be considered in cases where the ability to maintain and preserve the native hip anatomy is compromised.

Several surgical techniques and graft options have been proposed for labral reconstruction, including open surgical dislocation and arthroscopic techniques and autograft and allograft options (18, 20, 27, 33–36). The lead author prefers the arthroscopic front-to-back surgical technique for labral reconstruction with iliotibial band allograft. The technique described here differs from other arthroscopic techniques in that previous techniques fix the graft in the front and back first, followed by fixation in between. The success of that technique relies on creation of the perfectly sized graft, which can be challenging. The front-to-back technique described here allows the surgeon to make a graft that is 1–2 cm longer than necessary and cut excess graft after front-to-back fixation. The resulting graft is correct size, and the procedure is reproducible; however, it is important to note that the procedure is also technically demanding. Some tips for the “experienced hip arthroscopist” but “novice labral reconstructionist” are provided in Table S2 in Supplementary Material. Adequate training and practice in hip arthroscopy and labral reconstruction are necessary in order to ensure proficiency in placing the anchors in the most anterior and posterior position on the acetabular rim and being able to manage and appropriately fix the graft to obtain a perfect seal with the femoral head.

Long-term outcomes are necessary to determine the longevity of this procedure, but promising early outcomes of have been achieved (18, 20, 23–26, 38, 39). The published literature indicates few complications, improved subjective patient scores, and a low revision rate. Short-term improvement in patient symptomology and function were appreciated with both open and arthroscopic labral reconstruction. Labral repair remains an option in the young, healthy patient with healthy labral tissue; however, labral reconstruction should be considered in patients who do not meet these criteria.

Overall, labral reconstruction increases function, decreases pain, leads to a high level of patient satisfaction, and allows patients to return to activities of daily living and athletics. Labral reconstruction restores the original anatomy of the hip and has the potential to preserve the longevity of the hip joint. This technique is an important tool in the orthopedic surgeon’s arsenal for hip joint preservation.

## Conflict of Interest Statement

The authors declare that the research was conducted in the absence of any commercial or financial relationships that could be construed as a potential conflict of interest.

## Supplementary Material

The Supplementary Material for this article can be found online at http://journal.frontiersin.org/article/10.3389/fsurg.2015.00027

## References

[1] Glick JM Valone F III Safran MR. Hip arthroscopy: from the beginning to the future – an innovator’s perspective. Knee Surg Sports Traumatol Arthrosc (2014) 22:714–21. 10.1007/s00167-014-2859-y 24482213

[2] Burman MS Arthroscopy or the direct visualization of joints: an experimental cadaver study. 1931. Clin Orthop Relat Res (2001) 390:5–9. 10.1097/00003086-200109000-00003 11550876

[3] Ayeni OR Levy BA Musahl V Safran MR Current state-of-the-art of hip arthroscopy. Knee Surg Sports Traumatol Arthrosc (2014) 22:711–3. 10.1007/s00167-014-2866-z 24509832

[4] Reiman MP Goode AP Cook CE Holmich P Thorborg K. Diagnostic accuracy of clinical tests for the diagnosis of hip femoroacetabular impingement/labral tear: a systematic review with meta-analysis. Br J Sports Med (2014) 49:811. 10.1136/bjsports-2014-094302 25515771

[5] Krych AJ Kuzma SA Kovachevich R Hudgens JL Stuart MJ Levy BA. Modest mid-term outcomes after isolated arthroscopic debridement of acetabular labral tears. Knee Surg Sports Traumatol Arthrosc (2014) 22:763–7. 10.1007/s00167-014-2872-1 24493256

[6] McCarthy JC Noble PC Schuck MR Wright J Lee J. The Otto E. Aufranc award: the role of labral lesions to development of early degenerative hip disease. Clin Orthop Relat Res (2001) 393:25–37. 10.1097/00003086-200112000-00004 11764355

[7] Narvani AA Tsiridis E Kendall S Chaudhuri R Thomas P. A preliminary report on prevalence of acetabular labrum tears in sports patients with groin pain. Knee Surg Sports Traumatol Arthrosc (2003) 11:403–8. 10.1007/s00167-003-0390-7 12897984

[8] Ayeni OR Alradwan H de Sa D Philippon MJ. The hip labrum reconstruction: indications and outcomes – a systematic review. Knee Surg Sports Traumatol Arthrosc (2014) 22:737–43. 10.1007/s00167-013-2804-5 24318405

[9] Nepple JJ Philippon MJ Campbell KJ Dornan GJ Jansson KS LaPrade RF The hip fluid seal – part II: the effect of an acetabular labral tear, repair, resection, and reconstruction on hip stability to distraction. Knee Surg Sports Traumatol Arthrosc (2014) 22:730–6. 10.1007/s00167-014-2875-y 24509878

[10] Philippon MJ Nepple JJ Campbell KJ Dornan GJ Jansson KS LaPrade RF The hip fluid seal – part I: the effect of an acetabular labral tear, repair, resection, and reconstruction on hip fluid pressurization. Knee Surg Sports Traumatol Arthrosc (2014) 22:722–9. 10.1007/s00167-014-2874-z 24519614

[11] Ferguson SJ Bryant JT Ganz R Ito K. An in vitro investigation of the acetabular labral seal in hip joint mechanics. J Biomech (2003) 36:171–8. 10.1016/S0021-9290(02)00365-2 12547354

[12] Ferguson SJ Bryant JT Ganz R Ito K The influence of the acetabular labrum on hip joint cartilage consolidation: a poroelastic finite element model. J Biomech (2000) 33:953–60. 10.1016/S0021-9290(00)00042-7 10828325

[13] Crawford MJ Dy CJ Alexander JW Thompson M Schroder SJ Vega CE The 2007 Frank Stinchfield Award. The biomechanics of the hip labrum and the stability of the hip. Clin Orthop Relat Res (2007) 465:16–22. 10.1097/BLO.0b013e31815b181f 17906586

[14] Greaves LL Gilbart MK Yung AC Kozlowski P Wilson DR. Effect of acetabular labral tears, repair and resection on hip cartilage strain: a 7T MR study. J Biomech (2010) 43:858–63. 10.1016/j.jbiomech.2009.11.016 20015494

[15] Lee S Wuerz TH Shewman E McCormick FM Salata MJ Philippon MJ Labral reconstruction with iliotibial band autografts and semitendinosus allografts improves hip joint contact area and contact pressure: an in vitro analysis. Am J Sports Med (2015) 43:98–104. 10.1177/0363546514553089 25361860

[16] Larson CM Giveans MR. Arthroscopic debridement versus refixation of the acetabular labrum associated with femoroacetabular impingement. Arthroscopy (2009) 25:369–76. 10.1016/j.arthro.2008.12.014 19341923

[17] Larson CM Giveans MR Stone RM. Arthroscopic debridement versus refixation of the acetabular labrum associated with femoroacetabular impingement: mean 3.5-year follow-up. Am J Sports Med (2012) 40:1015–21. 10.1177/0363546511434578 22307078

[18] Philippon MJ Briggs KK Hay CJ Kuppersmith DA Dewing CB Huang MJ. Arthroscopic labral reconstruction in the hip using iliotibial band autograft: technique and early outcomes. Arthroscopy (2010) 26:750–6. 10.1016/j.arthro.2009.10.016 20511032

[19] Larson CM Giveans MR. Arthroscopic management of femoroacetabular impingement: early outcomes measures. Arthroscopy (2008) 24:540–6. 10.1016/j.arthro.2007.11.007 18442686

[20] Sierra RJ Trousdale RT. Labral reconstruction using the ligamentum teres capitis: report of a new technique. Clin Orthop Relat Res (2009) 467:753–9. 10.1007/s11999-008-0633-5 19048354 PMC2635467

[21] Cadet ER Chan AK Vorys GC Gardner T Yin B. Investigation of the preservation of the fluid seal effect in the repaired, partially resected, and reconstructed acetabular labrum in a cadaveric hip model. Am J Sports Med (2012) 40:2218–23. 10.1177/0363546512457645 22962293

[22] Ayeni OR Adamich J Farrokhyar F Simunovic N Crouch S Philippon MJ Surgical management of labral tears during femoroacetabular impingement surgery: a systematic review. Knee Surg Sports Traumatol Arthrosc (2014) 22:756–62. 10.1007/s00167-014-2886-8 24519616

[23] Walker JA Pagnotto M Trousdale RT Sierra RJ. Preliminary pain and function after labral reconstruction during femoroacetabular impingement surgery. Clin Orthop Relat Res (2012) 470:3414–20. 10.1007/s11999-012-2506-1 22864618 PMC3492611

[24] Boykin RE Patterson D Briggs KK Dee A Philippon MJ. Results of arthroscopic labral reconstruction of the hip in elite athletes. Am J Sports Med (2013) 41:2296–301. 10.1177/0363546513498058 23928321

[25] Geyer MR Philippon MJ Fagrelius TS Briggs KK. Acetabular labral reconstruction with an iliotibial band autograft: outcome and survivorship analysis at minimum 3-year follow-up. Am J Sports Med (2013) 41:1750–6. 10.1177/0363546513487311 23644149

[26] Matsuda DK Burchette RJ. Arthroscopic hip labral reconstruction with a gracilis autograft versus labral refixation: 2-year minimum outcomes. Am J Sports Med (2013) 41:980–7. 10.1177/0363546513482884 23548806

[27] Deshmane PP Kahlenberg CA Patel RM Han B Terry MA. All-arthroscopic iliotibial band autograft harvesting and labral reconstruction technique. Arthrosc Tech. (2013) 2:e15–9. 10.1016/j.eats.2012.10.001 23767003 PMC3678604

[28] Ejnisman L Philippon MJ Lertwanich P. Acetabular labral tears: diagnosis, repair, and a method for labral reconstruction. Clin Sports Med (2011) 30:317–29. 10.1016/j.csm.2010.12.006 21419958

[29] Bedi A Chen N Robertson W Kelly BT. The management of labral tears and femoroacetabular impingement of the hip in the young, active patient. Arthroscopy (2008) 24:1135–45. 10.1016/j.arthro.2008.06.001 19028166

[30] Ng VY Arora N Best TM Pan X Ellis TJ Efficacy of surgery for femoroacetabular impingement: a systematic review. Am J Sports Med (2010) 38:2337–45. 10.1177/0363546510365530 20489213

[31] Botser IB Smith TW Jr Nasser R Domb BG. Open surgical dislocation versus arthroscopy for femoroacetabular impingement: a comparison of clinical outcomes. Arthroscopy (2011) 27:270–8. 10.1016/j.arthro.2010.11.008 21266277

[32] Domb BG Stake CE Botser IB Jackson TJ Surgical dislocation of the hip versus arthroscopic treatment of femoroacetabular impingement: a prospective matched-pair study with average 2-year follow-up. Arthroscopy (2013) 29:1506–13. 10.1016/j.arthro.2013.06.010 23992988

[33] Domb BG Gupta A Stake CE Hammarstedt JE Redmond JM. Arthroscopic labral reconstruction of the hip using local capsular autograft. Arthrosc Tech. (2014) 3:e355–9. 10.1016/j.eats.2014.02.004 25126503 PMC4129983

[34] Matsuda DK. Arthroscopic labral reconstruction with gracilis autograft. Arthrosc Tech. (2012) 1:e15–21. 10.1016/j.eats.2011.12.001 23766969 PMC3678657

[35] Park SE Ko Y. Use of the quadriceps tendon in arthroscopic acetabular labral reconstruction: potential and benefits as an autograft option. Arthrosc Tech. (2013) 2:e217–9. 10.1016/j.eats.2013.02.003 24265987 PMC3834660

[36] White BJ Herzog MM Arthroscopic labral reconstruction of the hip using iliotibial band allograft and front-to-back fixation technique. Arthrosc Tech (2015) (in press). 10.1016/j.eats.2015.08.009 PMC4809662 27073784

[37] Ferro FP Philippon MJ Rasmussen MT Smith SD LaPrade RF Wijdicks CA Tensile properties of the human acetabular labrum and hip labral reconstruction grafts. Am J Sports Med (2015) 43:1222–7. 25660189 10.1177/0363546514568086

[38] White BJ Stapleford AB Hawkes TK Finger MJ Herzog MM Allograft use in arthroscopic labral reconstruction of the hip: minimum 2-year follow-up with front-to-back fixation technique. Arthroscopy (2015) (in press). 10.1016/j.arthro.2015.07.016 26422708

[39] Domb BG El Bitar YF Stake CE Trenga AP Jackson TJ Lindner D. Arthroscopic labral reconstruction is superior to segmental resection for irreparable labral tears in the hip: a matched-pair controlled study with minimum 2-year follow-up. Am J Sports Med (2014) 42:122–30. 10.1177/0363546513508256 24186974

