# Supplementary material for: Labral Reconstruction: When to Perform and How
Source: Front Surg. 2015 Jul 2;2:27. doi: 10.3389/fsurg.2015.00027 (PMC4489330; doi:10.3389/fsurg.2015.00027)
Supplement: Supplementary file 1 [file table_1.pdf]

Table S1. Indications and contraindications for arthroscopic labral reconstruction

Indications	Contraindications
- Irreparable labral tear<br>- Intrasubstance degeneration<br>- Labral tissue <2-3mm<br>- Labral tissue >8mm<br>- Previous labral surgery<br>- Rim ossification<br>- Severe pincer with lateral CE angle >45	- Age >60 years<br>- Joint space <50% of contralateral joint space<br>- Loss of 50% of hip rotation arc<br>- Significant loss of joint space on sagittal sequences on MRI
