# Supplementary material for: Labral Reconstruction: When to Perform and How
Source: Front Surg. 2015 Jul 2;2:27. doi: 10.3389/fsurg.2015.00027 (PMC4489330; doi:10.3389/fsurg.2015.00027)
Supplement: Supplementary file 2 [file table_2.pdf]

Table S2. Pearls and pitfalls for the "experienced hip arthroscopist" but "novice labral reconstructionist"

Pearls	Pitfalls
- Normal traction utilized<br>- Access to the anterior inferior joint is possible with ability to work through the peripheral compartment when not in traction<br>- Access for anchor placement and graft fixation is made using the distal portal and percutaneously through the anterior inferior capsule<br>- Anchor placement and graft shuttling occurs in traction through the distal accessory portal through the intact anterior inferior capsule<br>- 3 portals are required so that tension can be maintained on the graft when fixing it front-to-back<br>- Anterior acetabulum best visualized from anterior lateral portal<br>- Posterior acetabulum best visualized from anterior medial portal	- Excess traction should not be employed<br>- Anchor placement is critical – the graft is sensitive to eversion and the anchors must be placed perfectly, as close to the edge of the acetabulum as possible without penetrating subchondral bone<br>- Access to entire acetabulum is necessary, and surgeon must be comfortable placing anchors around entire acetabulum<br>- Important to preserve the anterior capsule<br>- Meticulous graft preparation is important so the graft is workable for a reasonable time within the joint without swelling or fraying
